# An in silico comparison of a novel CORA‐based cranial closing wedge ostectomy methodology with three other techniques

**DOI:** 10.1111/vsu.14277

**Published:** 2025-06-10

**Authors:** William H. R. Petchell, Anna R. Bostock, Alexander J. German, Andrew W. Tomlinson

**Affiliations:** ^1^ Small Animal Teaching Hospital University of Liverpool Neston UK; ^2^ Fusion Implants Liverpool UK; ^3^ Institute of Life Course & Medical Sciences University of Liverpool Liverpool UK

## Abstract

**Objective:**

To describe a CORA‐based cranial closing wedge ostectomy methodology (CCWO_CORA_) and to determine whether the CCWO_CORA_ achieves a more accurate and precise postoperative tibial plateau angle (TPA_POST_) than three previously described methods.

**Study design:**

In silico study.

**Sample population:**

Thirteen client‐owned dogs.

**Methods:**

Computed tomography (CT) scans of six Labrador retriever, six German shepherd, six Rottweiler, and six small‐breed dog (<10 kg) tibiae, originally acquired for unrelated purposes, were used for in silico planning and execution of the CCWO_CORA_ and previously described procedures. The TPA_POST_, tibial long axis shift, change in tibial length and wedge angle were recorded and a linear mixed‐effects model was used to compare differences amongst techniques.

**Results:**

The median TPA_POST_ for the CCWO_CORA_ method was 5.00° (range: 5.00–5.00°) across a variety of tibial morphologies, whereas all other methods showed greater variability. Differences in TPA_POST_ were evident amongst methods (*p* < .001) and breeds (*p* < .001).

**Conclusions:**

In silico, CCWO_CORA_ methodology always achieved the target TPA_POST_ due to its intrinsic geometric principles. As such, CCWO_CORA_ surgeries achieved a more accurate TPA_POST_ than previously described CCWO techniques.

**Clinical significance:**

The CCWO_CORA_ provides clinicians with a cranial closing‐wedge ostectomy methodology with entirely predictable TPA_POST_.

**Abbreviations**
3D: three dimensional
ACA‐CORA: angulation correction axis center of rotation of angulation
CBLO: CORA based leveling osteotomy
CCWO: cranial closing wedge ostectomy
CCWO_CORA_
: CORA‐based cranial closing wedge ostectomy
CORA: center of rotation of angulation
COV: coefficient of variation
CrCl: cranial cruciate ligament
CT: computed tomography
DICOM: digital imaging and communications in medicine
DMA: distal mechanical axis
FDR: false discovery rate
GSDs: German Shepherd Dogs
HSD: Tukey's Honestly Significant Difference
LBR: labrador retriever
LSM: least squares means
MAA: mechanical axis advancement
PJOL: proximal joint orientation line
PMA: proximal mechanical axis
Rot: Rottweiler
SMB: small breed dogs
tBL: transverse bisecting line
TPA: tibial plateau angle
TPA_POST_
: post operative tibial plateau angle
TPA_PRE_
: preoperative tibial plateau angle
TPLO: tibial plateau leveling osteotomy
ΔTL: change in tibial length

## 1 INTRODUCTION

Cranial closing wedge ostectomy (CCWO) was first described in 1984 as a method for eliminating cranial tibial translation in the cranial cruciate ligament (CrCL) deficient stifle.^1^ Numerous clinical, cadaveric, and in silico reports since this initial report have described adaptations and modifications of this technique.^2^, ^3^, ^4^, ^5^, ^6^, ^7^

The postoperative tibial plateau angles (TPA_POST_) of tibial plateau leveling osteotomy (TPLO) can be selected accurately as the tibial plateau is rotated until the desired TPA_POST_ is achieved.^8^ As they are not geometrically founded, existing CCWO planning methodologies do not enable calculation of a precise TPA_POST_ following acute surgical correction. Inaccurate correction of TPA can lead to unsatisfactory outcomes with CCWO.^9^ Ex vivo, a CCWO that achieves a TPA_POST_ between 4° and 6° satisfactorily eliminates cranial tibial thrust, whereas a TPA_POST_ < 4° results in caudal tibial subluxation.^10^ Furthermore, TPA_POST_ > 6° may not adequately eliminate cranial tibial thrust.^10^ An explanation for the inherent inaccuracies observed with existing CCWO methodologies is that tibial mechanical axis shift is neither quantifiable nor predictable. Tibial mechanical axis shift and TPA_POST_ are linked intrinsically, therefore, failure to control or account for tibial mechanical axis shift leads to unreliable TPA_POST_.^11^

Center of rotation of angulation (CORA) principles were described in 1994 as a method of correcting angular limb deformities in human patients;^12^ these principles have been utilized by veterinary surgeons to correct angular limb deformity.^13^, ^14^, ^15^ The principles are integral to the CORA based leveling osteotomy (CBLO) technique described for the stabilization of the CrCL‐deficient canine stifle.^16^ The authors believed that, if CORA principles were applied to CCWO methodology, precise quantification of mechanical axis shift would occur and produce a more accurate and predictable correction, overcoming the limitations of previous CCWO methodologies. The aims of this study were therefore: (1) to describe a CORA‐based CCWO methodology (CCWO_CORA_) and (2) to conduct an in silico study to determine whether the CCWO_CORA_ achieves more accurate and repeatable TPA_POST_ compared with three previously described techniques.^4^, ^5^, ^6^ It was hypothesized that: (1) the CCWO_CORA_ technique would always achieve the desired TPA_POST_, and (2) that the CCWO_CORA_ technique would achieve more accurate and predictable TPA_POST_ across a range of tibial morphologies.

## 2 MATERIALS AND METHODS

### 2.1 Study population, ethics and eligibility criteria

Prior to study commencement, approval was granted by the University of Liverpool institutional ethics committee (approval number: VREC1379). Archived computed tomography (CT) scans obtained between March 2008 and April 2022 of the pelvic limbs of Labrador retriever (LBR), German shepherd dogs (GSDs), Rottweilers, and small‐breed dogs (<10 kg) were reviewed retrospectively. To be eligible, scans of the complete tibia and tarsus had to be available. Dogs were not eligible if there was any evidence of tibial pathology. Images were obtained using an 80‐slice multidetector unit (Canon Medical Systems. Aquilion Lightning CT Scanner. Otawara, Japan: Canon Medical Systems Corporation) with a slice thickness of 0.5 mm. Digital Imaging and Communications in Medicine (DICOM) files were imported into medical image processing software (Materialise. Mimics Inovation Suite, version 25.0. Leuven, Belgium: Materialise; 2022) to create three‐dimensional (3D) tibial bone models; these models were exported to computer‐aided design software (Materialise. 3‐matic, version 17.0. Leuven, Belgium: Materialise; 2023) for in silico surgical planning and execution.

### 2.2 Preoperative planning

**FIGURE 1:**
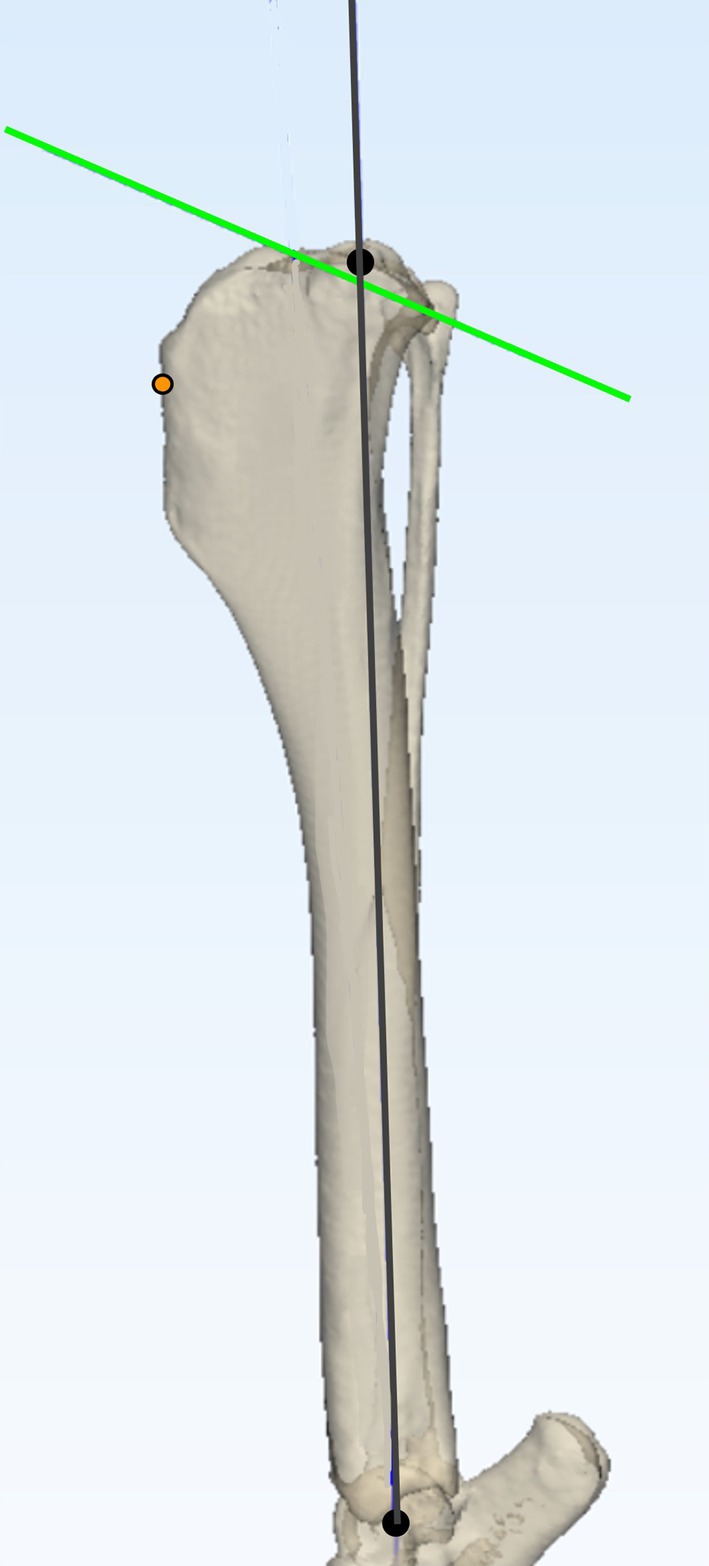
Landmark and axis planning on a three‐dimensional tibial bone model. Three anatomical landmarks were agreed by consensus: (1) a point 5 mm distal to the insertion of the patellar tendon on the cranial aspect of the tibial crest (orange circle); (2) the center of the talus (distal black circle); and (3) the base of the intercondylar eminences (proximal black circle). The mechanical axis (gray line) was plotted between the center of the talus and the base of the intercondylar eminences. Points at the cranial and caudal aspect of the tibial plateau were identified by consensus. These points were joined to determine the proximal joint orientation line (green line).

The tibial mechanical axis and proximal joint orientation lines (PJOL) were plotted on each tibia.^17^ A point 5 mm distal to the insertion of the patellar tendon was marked. The center of the talus, the base of the medial malleolus, and the base of the intercondylar eminences were marked to allow consistent calculation of postoperative mechanical axis advancement (MAA) and change in tibial length (*Δ*TL) (Figure 1). These marks remained unchanged for each surgery to ensure consistency in planning, preoperative and postoperative measurements across techniques. Preoperative tibial plateau angle (TPA_PRE_) was determined as described previously.^18^ The distance between the base of the intercondylar eminences and base of the medial malleolus was calculated and recorded as the tibial length.

### 2.3 Terreros and Daye (CCWO_TPA_ _−5_) & Frederick and Cross (CCWO_TPA_ ) methodologies

**FIGURE 2:**
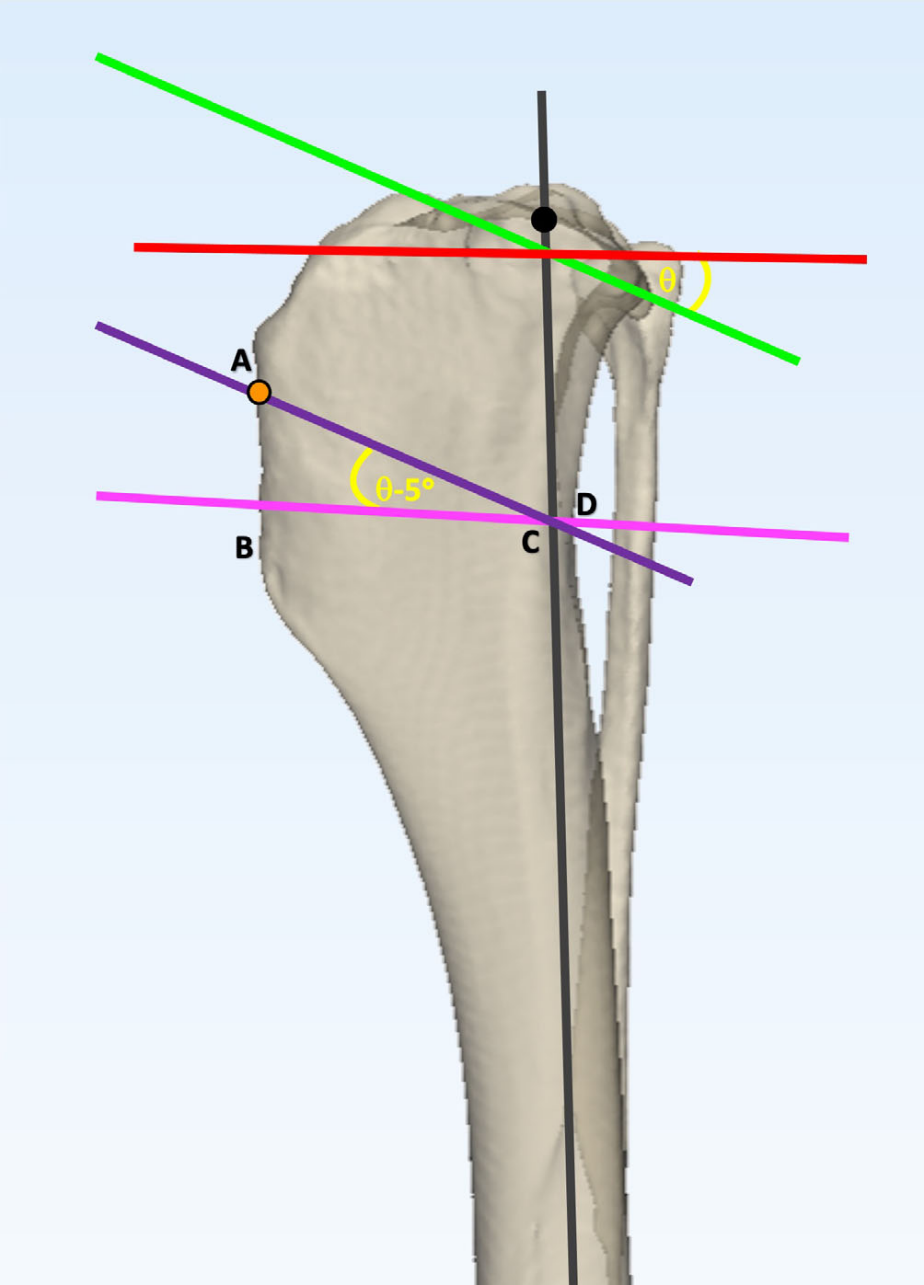
Planning of the CCWO_TPA‐5_ methodology on a three‐dimensional tibial bone model. The mechanical axis (gray line) was plotted between the center of the talus and the base of the intercondylar eminences. Points at the cranial and caudal aspect of the tibial plateau were agreed by consensus. These points were joined to determine the proximal joint orientation line (PJOL) (green line). A line was plotted at 90° to the mechanical axis at the level of its intersection of the tibial PJOL (red line) and the tibial plateau angle (TPA) (θ) was measured. The proximal osteotomy line (AC) (purple line) was plotted parallel to the PJOL starting at a point 5 mm distal to the point of insertion of the patellar tendon (orange circle). The second osteotomy line (BD) (pink line) was planned at 85° to the mechanical axis, thus creating a wedge angle equal to TPA − 5°. The distal osteotomy line was moved either proximally or distally, to ensure the line AC was the same length as BD.

**FIGURE 3:**
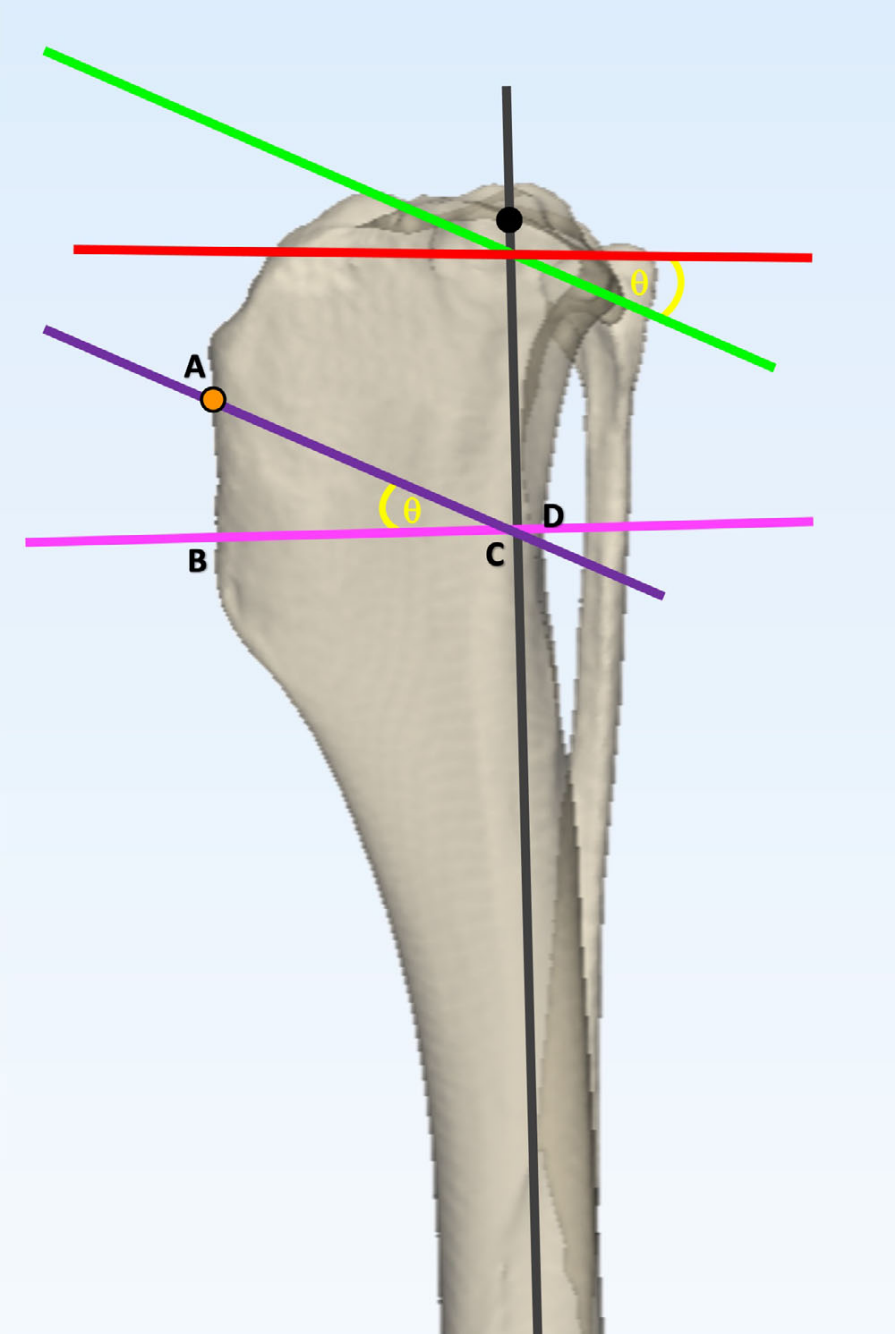
Planning of the CCWO_TPA_ methodology on a three‐dimensional tibial bone model. The mechanical axis (gray line) was plotted between the center of the talus and the base of the intercondylar eminences. Points at the cranial and caudal aspect of the tibial plateau were agreed by consensus. These points were joined to determine the proximal joint oreientation line (PJOL) (green line). A line was plotted at 90° to the mechanical axis at the level of its intersection with the PJOL (red line) and the tibial plateau angle (TPA) (θ) was measured. The proximal osteotomy line (AC) (purple line) was plotted parallel to the PJOL starting at the point 5 mm distal to the point of insertion of the patellar tendon (orange circle). The second osteotomy line (BD) (pink line) was planned at 90° to the mechanical axis thus creating a wedge angle equal to the TPA (θ). Line BD was moved either proximally or distally to ensure the line AC was the same length as BD.

CCWO_TPA−5_ ^6^ (Figure 2) and CCWO_TPA_ ^5^ (Figure 3) surgeries were planned as described previously, ensuring that the proximal osteotomy exited the cranial tibial cortex 5 mm below the insertion of the patellar tendon.

### 2.4 Oxley methodology (CCWO_ISO_ )

**FIGURE 4:**
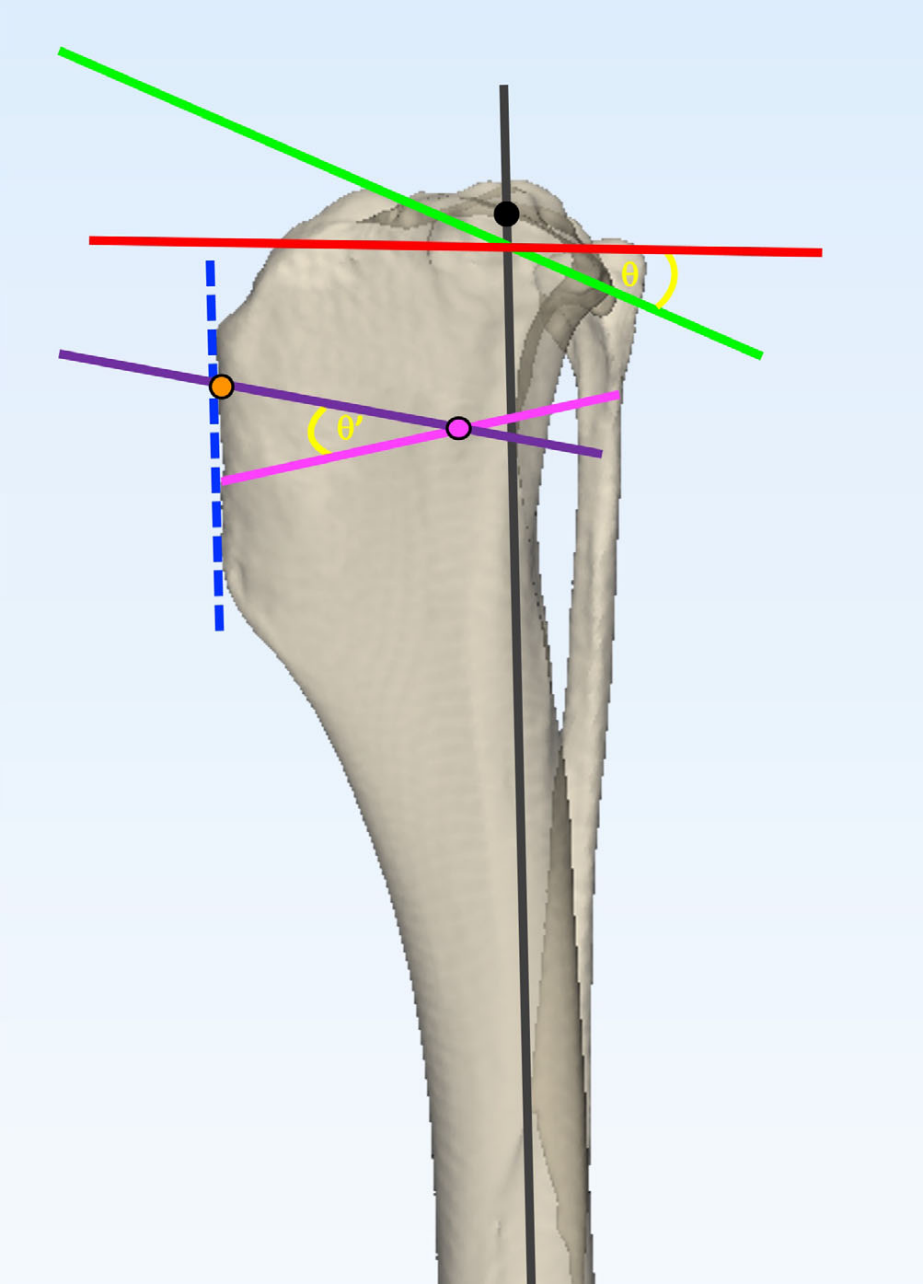
Planning of the CCWO_ISO_ methodology on a three‐dimensional tibial bone model. The mechanical axis (gray line) was plotted between the center of the talus and the base of the intercondylar eminences. Points at the cranial and caudal aspect of the tibial plateau were agreed by consensus. These points were joined to determine the proximal joint orientation line (PJOL) (green line). A line was plotted at 90° to the mechanical axis at the level of its intersection with the PJOL (red line) and the tibial plateau angle (θ) was measured. The cranial cortex of the tibial tuberosity was plotted (blue dashed line). An isosceles triangle was plotted perpendicular to the cranial cortex of the tibial tuberosity with the wedge angle (θ′) determined as follows: TPA under 20°: θ′ = TPA‐5°, TPA between 21 and 25°: θ′ = TPA‐4°, TPA between 26 and 30°: θ′ = TPA‐3° and TPA between 31 and 35°: θ′ = TPA‐2°. The apex of the wedge (pink circle) was located at 75% of the width of the tibia along the proximal osteotomy line (purple line). The proximal osteotomy was plotted so it exited the cranial cortex of the tibia 5mm distal to the point of insertion of the patellar tendon (orange circle). The distal osteotomy (pink line) was planned so that it passed through the intended apex of the wedge at the desired wedge angle relative to the proximal osteotomy.

The CCWO_ISO_ ^4^ surgeries were planned as previously described with minor modifications to maintain repeatability across tibial morphologies (Figure 4). A line was plotted from the most cranial aspect of the tibial tuberosity to the most cranial aspect of the tibial crest. This line became the base of an isosceles triangle. The isosceles triangle was plotted with the proximal osteotomy exiting 5 mm distal to the insertion of the patellar tendon. The apex of the isosceles triangle was positioned 75% along the length of the proximal osteotomy.

### 2.5 CCWO_CORA_ methodology

**FIGURE 5:**
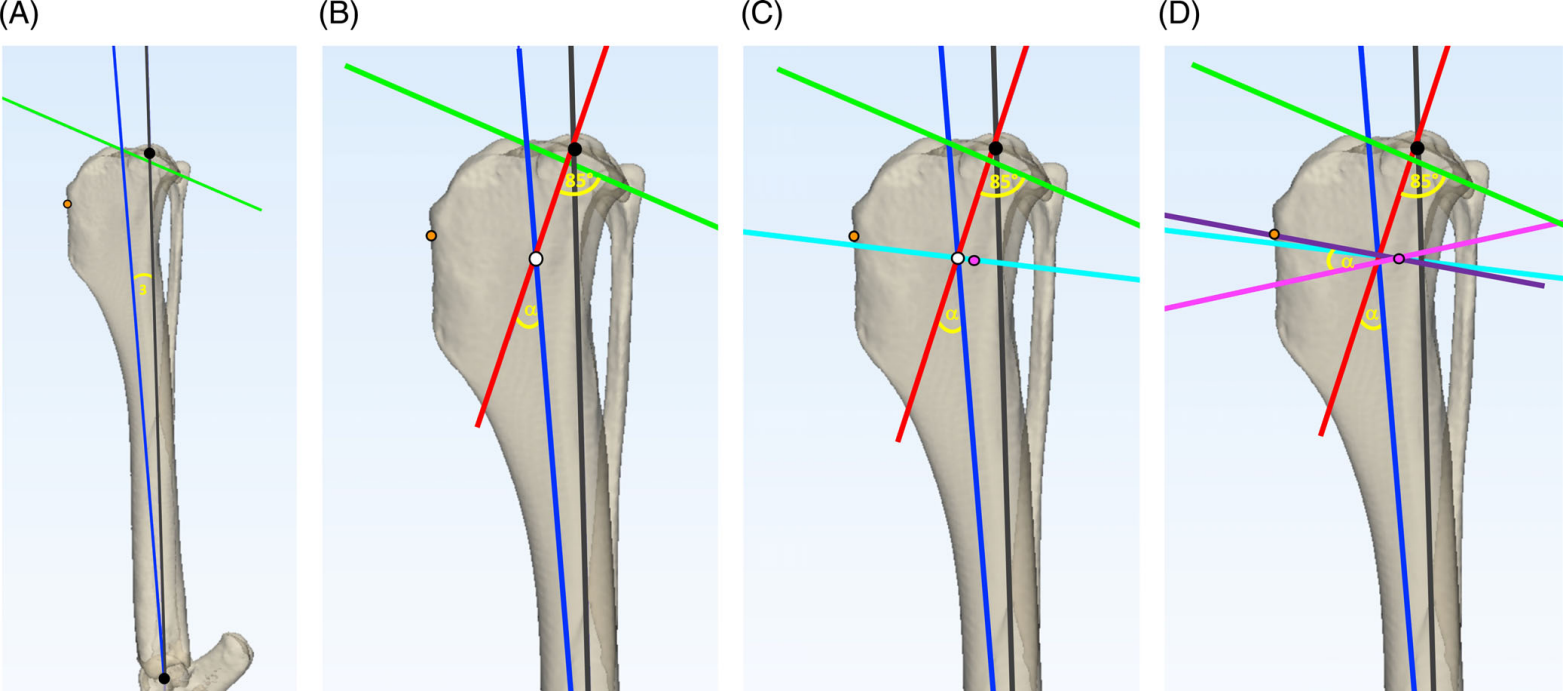
Planning of the CCWO_CORA_ methodology on a three‐dimensional tibial bone model. The mechanical axis (gray line) was plotted between the center of the talus and the base of the intercondylar eminences (black circles). Points at the cranial and caudal aspect of the tibial plateau were agreed by consensus and joined to determine the proximal joint orientation line (PJOL) (green line). The mechanical axis was advanced cranially by 3° (blue line) to create a distal mechanical axis (DMA) (A). The proximal mechanical axis (PMA) was plotted 5° caudal to a line perpendicular to the PJOL (red line). The intersection of the DMA and PMA determined the location (white circle) and magnitude (*α*) of the CORA (B). The transverse bisecting line (light blue line) and an angulation correction axis‐CORA (ACA‐CORA) 75% along this line was plotted (pink circle) (C). The cranial closing osteotomies were then planned. The proximal osteotomy (purple line) originated at the ACA‐CORA (pink circle) and exited at the mark 5 mm distal to the insertion of the patellar tendon (orange circle). The distal osteotomy (pink line) originated at the ACA‐CORA and was plotted at an angle equal to *α* (D).

**FIGURE 6:**
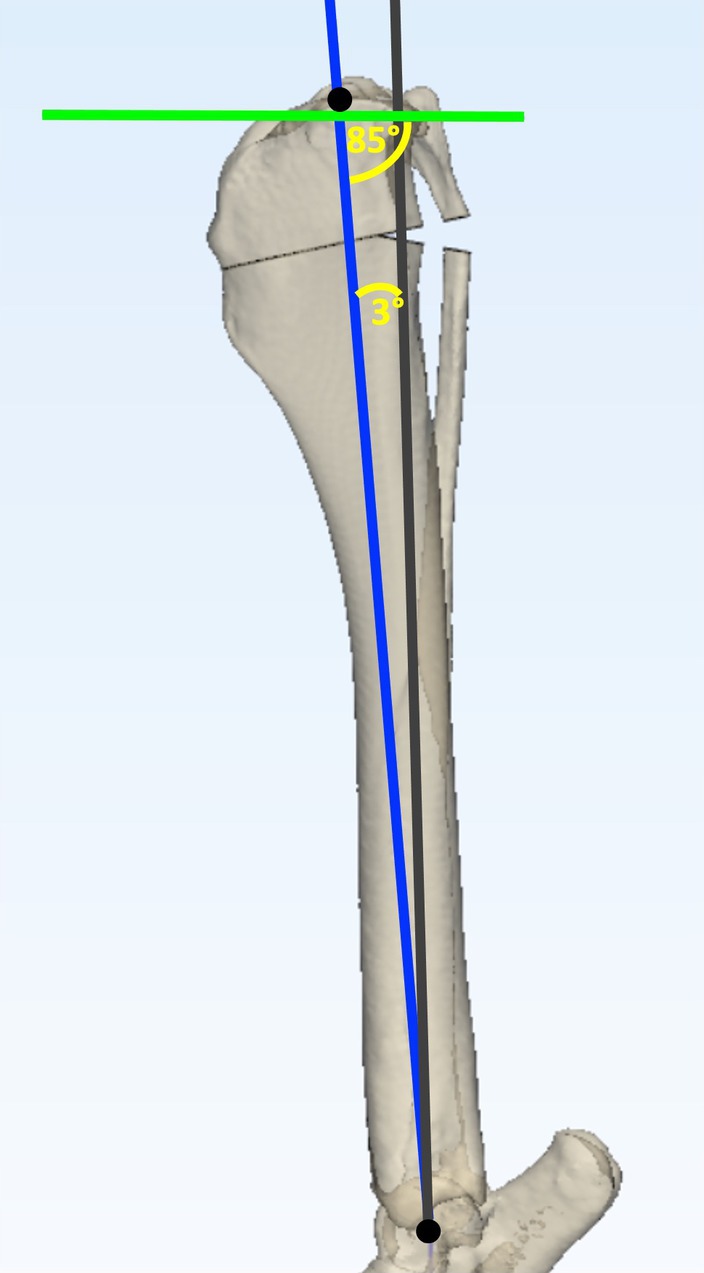
Execution of planned CCWO_CORA_ surgery on a three‐dimensional tibial bone model. The gray line represents the original mechanical axis of the tibia. The distal mechanical axis (blue line), which was advanced by 3°, now represents the postoperative mechanical axis. The preoperatively determined points at the cranial and caudal aspects of the tibial plateau were connected to determine the new proximal joint orientation line (green line). The mechanical caudal proximal tibial angle is exactly 85°.

The tibial mechanical axis was advanced by rotating the mechanical axis cranially by 3° from the center of the talus generating a distal mechanical axis (DMA) (Figure 5A). Next, the proximal mechanical axis (PMA) was plotted 5° caudal to a line perpendicular to the PJOL. The intersection of the DMA and PMA determined the location and magnitude (*α*) of the CORA (Figure 5B). The transverse bisecting line (tBL) was plotted and an angulation correction axis‐CORA (ACA‐CORA) 75% along this line was marked (Figure 5C). A cranial closing wedge was plotted with the proximal osteotomy originating at the ACA‐CORA and exiting 5 mm distal to the insertion of the patellar tendon. The distal osteotomy originated at the ACA‐CORA and was plotted at an angle equal to α (Figure 5D). The wedge ostectomy was executed in silico and the proximal fragment rotated about the ACA‐CORA until the bone fragments contacted; the cranial cortices were not aligned (Figure 6).

### 2.6 CCWO_CORA_ methodology small‐breed group

**FIGURE 7:**
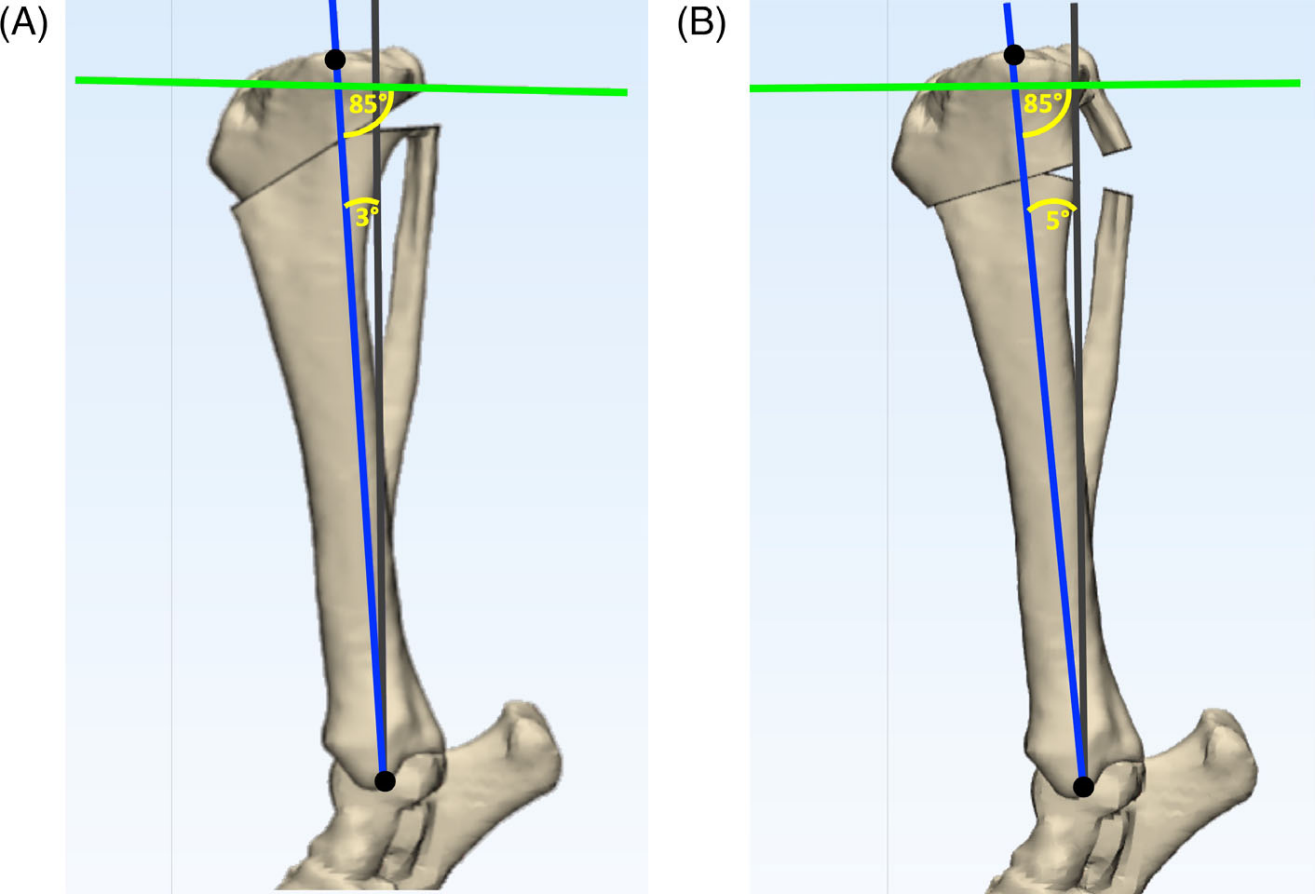
The effects of different mechanical axis advancement on small‐breed dogs undergoing CCWO_CORA_ on a three‐dimensional tibial bone model. The gray line represents the original mechanical axis of the tibia. The preoperatively determined points at the cranial and caudal aspects of the tibial plateau were connected to determine the proximal joint orientation line (PJOL) (green line). The distal mechanical axis was advanced by 3° in (A) and by 5° in (B), both of which resulted in a postoperative tibial plateau angle of exactly 5°. In small‐breed dogs with steeper tibial plateau angles, a greater distal mechanical axis advancement results in a larger proximal bone fragment.

In the small‐breed group, a CCWO_CORA_ surgery was completed as outlined above with a MAA of 3° (CCWO_CORA3_). A second CCWO_CORA_ surgery was completed on each tibia. The only difference for the second surgery was that step 1 was modified so that a MAA of 5° was plotted (CCWO_CORA5_) (Figure 7).

### 2.7 Postoperative measurements

Postoperative TPA_POST_, MAA, *Δ*TL and wedge ostectomy angle were recorded for each dog. TPA_POST_ was measured as per the TPA_PRE_ using the same landmarks. The MAA was measured as the angle between the preoperative tibial mechanical axis and the postoperative tibial mechanical axis. Postoperative tibial length was calculated by measuring the length between the mark at the base of the intercondylar eminence and the mark at the center of the medial malleolus on the postoperative tibiae (Figures 6 and 7). The *Δ*TL was calculated by dividing preoperative tibial length by postoperative tibial length and multiplying by 100. Wedge ostectomy angles were equal to *α*.

### 2.8 Data handling and statistical analysis

The primary outcome was TPA_POST_, with all other postoperative measurements being secondary outcomes. Based on a two‐tailed, a priori power analysis, at least 17 CCWO_CORA_, and 68 in silico surgeries were necessary to ensure a power of at least 95% (α = 0.05, effect size = 0.8, paired *t*‐test). Data are reported as median (range), unless indicated, and the threshold for statistical significance was *p* < .05, with all analyses being two sided.

Statistical analysis was performed using an online, open‐access statistical language and environment (R version 4.3.1),^19^ along with several additional packages.^20^, ^21^, ^22^, ^23^, ^24^, ^25^, ^26^, ^27^, ^28^, ^29^, ^30^, ^31^ The Kruskal–Wallis test, with Dunn's post hoc test, was used to compare differences in TPA_PRE_ amongst breeds. Linear mixed‐effects models were used to compare differences in postoperative measurements (TPA_POST_, MAA, ∆ TL and wedge angle) among methods. Dog and leg (left or right) were included as random effects, with leg nested within dog. Method, breed, and the interaction between method and breed were included as fixed effects. The distribution of the residuals from all models were tested for skewness and normality by visual inspection and with the Shapiro–Wilk test, and the possibility of heteroscedasticity was assessed by visually inspecting a plot of residuals against predictions and with Levene's test. The nlme package version 3.1–66^21^ was used for these analyses, because unequal variance amongst groups could be accounted for (e.g., in models where there was evidence of heteroscedasticity). Pairwise contrasts were calculated both between methods, and amongst breeds within each method. Tukey's honestly significant difference (HSD) test was used for these comparisons, enabling *p* values to be corrected for false discovery rate (FDR). The results of these analyses are reported as least squares means (LSM) and their 95% confidence interval (95% CI). Cohen's *d* was calculated to determine the effect size using the “eff_size” function of the “emmeans” package,^22^ interpreted according to Lakens:^23^ *d* < 0.1 is classified as very small, 0.1 ≤ *d* < 0.3 is small, 0.3 ≤ *d* < 0.5 is medium, and *d* ≥ 0.5 is large.

Finally, the Wilcoxon signed‐ranks test was used to compare the results in the small‐breed group for CCWO_CORA_ methodology utilizing different angles of advancement (3° MAA and 5° MAA). Effect size was determined using the rank‐biserial correlation and interpreted as described by Funder and Ozer:^24^ <0.05, tiny; 0.05–0.10, very small; 0.10–0.20, small; 0.20–0.30, medium; 0.30–0.40, large; >0.40 very large.

## 3 RESULTS

### 3.1 Study dogs

The scans of three LBRs (*n* = 6 tibia), three Rottweilers (6), three GSDs (6), two Yorkshire terriers (3) and two West Highland white terriers (3) met the inclusion criteria, totaling 24 tibiae. The mean age at CT acquisition was 28 months for LBRs, 29 months for Rottweilers, 42 months for GSDs, and 65 months for small‐breed dogs. Mean ± standard deviation (SD) TPA_PRE_ was 24.15 ± 2.30°, 24.10 ± 1.75°, 25.00 ± 0.34° and 33.28 ± 2.17° for LBR, Rottweilers, GSDs and small‐breed dogs, respectively.

### 3.2 Postoperative TPA

**FIGURE 8:**
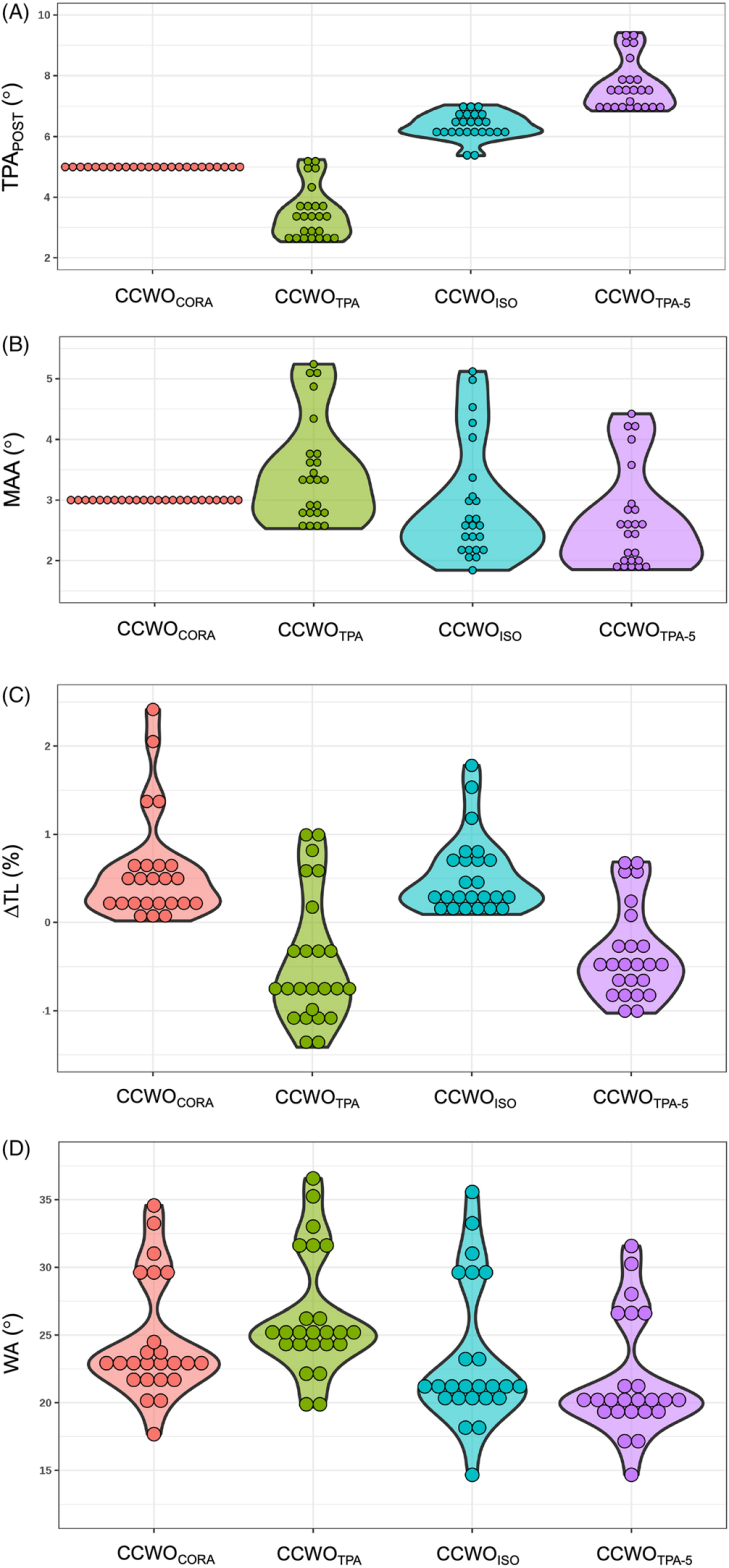
Violin plot demonstrating the absolute postoperative tibial plateau angles (TPA_POST_), mechanical axis advancement (MAA), percentage change between preoperative and postoperative tibial length (∆ TL), and wedge angle for CCWO_CORA_, CCWO_ISO_, CCWO_TPA‐5_, and CCWO_TPA_ planned surgeries. The circles represent the absolute postoperative TPA_POST_ (A), MAA (B), ∆ TL (C) and wedge angle (D). In (A), the shaded area around the individual circles represents the kernel density estimation to show the distribution shape of the data whereas the shaded area in Figure 8B–D is a compact display of the continuous distribution, where the width is proportionate to the data density in each region. Mean TPA_POST_ varied amongst planning methods (*p* < .001), and variability was least for the CCWO_CORA_ method (Levene's test, *p* < .001). Mean MAA varied amongst planning methods (*p* < .001), and variability was least for the CCWO_CORA_ method (Levene's test, *p* < .001). Mean *Δ*TL varied amongst planning methods (*p* < .001), but there was no difference in variability amongst methods (Levene's test, *p* = .331). Mean wedge angle varied amongst planning methods (*p* < .001) but there was no difference in variability amongst methods (Levene's test, *p* = .928).

Differences in TPA_POST_ amongst methods are shown in Figure 8A. The median (range) TPA_POST_ for all CCWO_CORA_ surgeries was 5.00° (range: 5.00° to 5.00°); it was 3.35° (range: 2.53° to 5.24°) for all CCWO_TPA_ surgeries; 6.33° (range: 5.37° to 7.04°) for all CCWO_ISO_ surgeries, and 7.52° (range 6.85° to 9.42°) for all CCWO_TPA‐5_ surgeries. Variability was less for the CCWO_CORA_ method than for all other methods (Levene's test, *p* < .001; Table 1). In the linear mixed‐effects model, TPA_POST_ varied by planning method (*p* < .001, Cohen's *d* 1.00, a large effect), with post hoc comparisons demonstrating that all methods differed from one another (*p* < .001; Table 2). The TPA_POST_ also varied amongst methods within each breed (*p* < .05) (Table 3), except for the comparison of CCWO_CORA_ with CCWO_TPA_ in small‐breed dogs (*p* = .331).

**TABLE 1:** Coefficients of variations of measurements made in cranial closing wedge ostectomy planning.

CCWO Method	TPA_POST_ (%)	MAA (%)	*Δ* TL (%)	Wedge angle (%)
CCWO_CORA_	0.0	0.0	103.0	17.7
CCWO_ISO_	6.8	32.7	81.7	22.7
CCWO_TPA_	24.9	24.9	171.1	17.0
CCWO_TPA‐5_	10.7	30.9	156.8	19.8

*Note*: The coefficients of variation for TPA_POST_, MAA, *Δ*TL, and wedge angle for all surgeries performed using the four CCWO techniques studied.

Abbreviations: CCWO, Cranial closing wedge ostectomy; MAA, mechanical axis advancement; *Δ*TL, change in tibial length; TPA_POST_, precise postoperative tibial plateau angle.

**TABLE 2:** Least squares means by method from linear mixed‐effects models.

CCWO method	TPA_POST_ (°)	MAA (°)	*Δ* TL (%)	Wedge angle (°)
CCWO_CORA_	4.96^a^ (4.59, 5.33)	2.96^a^ (2.58, 3.34)	+0.58^a^ (+0.33, +0.83)	24.5^a^ (23.4, 25.6)
CCWO_ISO_	6.31^b^ (5.96, 6.65)	2.89^a^ (2.56, 3.22)	+0.52^a^ (+0.27, +0.77)	23.1^b^ (22.1, 24.2)
CCWO_TPA_	3.44^c^ (3.13, 3.75)	3.44^b^ (3.11, 3.76)	−0.45^b^ (−0.70, −0.20)	26.4^c^ (25.3, 27.4)
CCWO_TPA‐5_	7.63^d^ (7.33, 7.94)	2.63^c^ (2.31, 2.95)	−0.36^c^ (−0.61, −0.11)	21.6^d^ (20.5, 22.7)

*Note*: Results reported are least squares means and 95% confidence intervals derived from the linear mixed‐effects models. For each variable, methods that differed at *p* < .05 are shown by different superscript letters.

Abbreviations: CCWO, Cranial closing wedge ostectomy; MAA, mechanical axis advancement; *Δ*TL, change in tibial length; TPA_POST_, precise postoperative tibial plateau angle.

**TABLE 3:** Least squares means by method within breed from linear mixed‐effects models.

CCWO Method	TPA_POST_ (°)				Mechanical axis advancement (°)				*Δ* Tibial length (%)				Wedge angle (°)			
	LBR	GSD	ROT	SMB	LBR	GSD	ROT	SMB	LBR	GSD	ROT	SMB	LBR	GSD	ROT	SMB
CCWO_CORA_	**5.08** ^**a**^	**5.05** ^**a**^	**5.08** ^**a**^	**4.64** ^**a**^	**3.00** ^**a**^	**3.00** ^**ab**^	**3.00** ^**a**^	**2.84** ^**a**^	**+0.41** ^**a**^	**+0.32** ^**a**^	**+0.29** ^**a**^	**+1.29** ^**a**^	**21.8** ^**a**^	**23.0** ^**a**^	**22.1** ^**a**^	**31.3** ^**a**^
	(4.31, 5.85)	(4.30, 5.81)	(4.31, 5.86)	(3.84, 5.43)	(2.21, 3.79)	(2.24, 3.76)	(2.21, 3.79)	(2.12, 3.56)	(+0.10, +0.93)	(−0.18, +0.82)	(−0.23, +0.80)	(+0.83, +1.75)	(19.6, 24.1)	(20.8, 25.1)	(19.9, 24.3)	(29.0, 33.1)
CCWO_ISO_	**6.49** ^**b**^	**6.54** ^**b**^	**6.35** ^**b**^	**5.85** ^**a**^	**2.41** ^**a**^	**2.49** ^**a**^	**2.43** ^**a**^	**4.22** ^**b**^	**+0.37** ^**a**^	**+0.38** ^**a**^	**+0.41** ^**a**^	**+0.93** ^**b**^	**20.1** ^**b**^	**21.0** ^**b**^	**20.3** ^**b**^	**31.4** ^**a**^
	(5.77, 7.22)	(5.84, 7.25)	(5.62, 7.07)	(5.10, 6.60)	(1.73, 3.09)	(1.84, 3.15)	(1.75, 3.11)	(3.62, 4.82)	(−0.15, +0.89)	(−0.12, +0.88)	(−0.11, +0.92)	(+0.08, +1.00)	(17.9, 22.4)	(18.8, 23.1)	(18.0, 22.5)	(29.1, 33.3)
CCWO_TPA_	**3.14** ^**c**^	**3.21** ^**c**^	**3.10** ^**c**^	**4.31** ^**b**^	**3.05** ^**b**^	**3.16** ^**b**^	**3.02** ^**b**^	**4.52** ^**c**^	**−0.58** ^**b**^	**−0.92** ^**b**^	**−0.83** ^**b**^	**0.54** ^**c**^	**23.3** ^**c**^	**25.0** ^**c**^	**24.1** ^**c**^	**33.3** ^**b**^
	(2.48, 3.79)	(2.58, 3.85)	(2.44, 3.76)	(3.63, 5.00)	(2.38, 3.73)	(2.51, 3.81)	(2.34, 3.69)	(3.93, 5.11)	(−1.10, −0.07)	(−1.41, −0.42)	(−1.35, −0.32)	(+0.08, +1.00)	(21.1, 25.5)	22.8, 27.1	(21.9, 26.3)	(31.0, 35.1)
CCWO_TPA‐5_	**7.30** ^**d**^	**7.38** ^**d**^	**7.34** ^**d**^	**8.50** ^**c**^	**2.22** ^**c**^	**2.33** ^**c**^	**2.25** ^**c**^	**3.71** ^**d**^	**−0.40** ^**b**^	**−0.72** ^**b**^	**−0.65** ^**b**^	**0.34** ^**c**^	**19.1** ^**d**^	**20.0** ^**d**^	**19.1** ^**d**^	**28.3** ^**c**^
	(6.66, 7.95)	(6.76, 8.01)	(6.69, 7.99)	7 (.82, 9.18)	(1.56, 2.89)	(1.69, 2.97)	(1.59, 2.92)	(3.13, 4.29)	(−0.92, +0.11)	(−1.22, −0.22)	(−1.17, −0.14)	(−0.12, +0.80)	(16.9, 21.4)	(17.8, 22.1)	(16.9, 21.3)	(26.0, 30.1)

*Note*: Results reported are least squares means, and 95% confidence intervals derived from the linear mixed‐effects models. For each variable, methods that differed within breeds at *p* < .05 are shown by different superscript letters.

Abbreviations: CCWO, Cranial closing wedge ostectomy; GSD, German shepherd dogs; LBR, Labrador retriever; MAA, mechanical axis advancement; *Δ*TL, change in tibial length; ROT, Rottweiler; SMB, Small Breed Dogs; TPA_POST_, precise postoperative tibial plateau angle.

### 3.3 Mechanical axis advancement

Differences in MAA amongst methods are shown in Figure 8B. Median (range) mechanical axis advancement for CCWO_CORA_ surgeries was 3.00° (range: 3.00° to 3.00°); it was 3.35° (range: 2.53° to 5.24°) for CCWO_TPA_ surgeries, 2.61° (range: 1.84° to 5.12°) for CCWO_ISO_ surgeries, and 2.52° (range: 1.85° to 4.42°) for CCWO_TPA‐5_ surgeries. Variability was less for the CCWO_CORA_ method than for all other methods (Levene's test, *p* < .001; Table 1). In the linear mixed‐effects model, MAA varied by planning method (*p* < .001, Cohen's *d* 1.00, a large effect), with *post hoc* comparisons demonstrating that MAA for all methods differed from one another (CCWO_CORA_ and CCWO_TPA‐5_ *p* = .004; all other contrasts *p* < .001; Table 1), except between CCWO_CORA_ and CCWO_ISO_ (*p* = .884). For most comparisons, MAA also varied amongst methods within each breed (*p* < .05) (Table 3), except between CCWO_CORA_ and CCWO_TPA_ in LBR (*p* = .992), rottweilers (*p* = .999) and GSDs (*p* = .839), and between CCWO_CORA_ and CCWO_ISO_ in GSDs (*p* = .053).

### 3.4 Percentage change in tibial length (*Δ* TL)

Figure 8C shows differences in *Δ*TL amongst methods. Median (range) *Δ*TL for CCWO_CORA_ surgeries was +0.44% (range: +0.02% to +2.42%), −0.71% (range: −1.41% to +1.00%) for CCWO_TPA_ surgeries, +0.33% (range: +0.09% to +1.78%) for CCWO_ISO_ surgeries and −0.48% (range: −1.03% to +0.67%) for CCWO_TPA‐5_ surgeries. There was no difference in variability amongst methods (Levene's test, *p* = .331; Table 1). In the linear mixed‐effects model, *Δ*TL varied by planning method (*p* < .001, Cohen's *d* 1.00, a large effect), with *post hoc* comparisons demonstrating that *Δ*TL for all methods differed at *p* < .001 (Table 1), except between CCWO_CORA_ and CCWO_ISO_ (*p* = .796), and between CCWO_TPA_ and CCWO_TPA‐5_ (*p* = .498). For most comparisons, *Δ*TL also varied amongst methods within each breed (*p* < .05) (Table 3), except between CCWO_TPA_ and CCWO_TPA‐5_ in all breeds (GSDs *p* = .421; LBR *p* = .482, Rottweilers *p* = .487, small‐breed dogs *p* = .396), and for CCWO_CORA_ and CCWO_ISO_ in GSDs (*p* = .962), LBR (*p* = .986), and Rottweilers (*p* = .786).

### 3.5 Wedge angle

Differences in wedge angle amongst methods are shown in Figure 8D. Median (range) wedge angles for CCWO_CORA_ surgeries were 23.1° (range: 17.7° to 34.6°), 25.1° (range: 19.7° to 36.6°) for CCWO_TPA_ surgeries, 21.1° (range: 14.7° to 35.6°) for CCWO_ISO_ surgeries, and 20.1° (range: 14.7° to 31.6°) for CCWO_TPA‐5_ surgeries. There was no difference in variability amongst methods (Levene's test, *p* = .928; Table 1). In the linear mixed‐effects model, wedge angle varied by planning method (*p* < .001, Cohen's *d* 1.00, a large effect), with *post hoc* comparisons demonstrating that wedge angle for all methods differed from one another (*p* < .001; Table 2). Wedge angle also varied amongst method within all breeds (*p* < .002 for all; Table 3).

### 3.6 Comparison of CCWO_CORA3_ and CCWO_CORA5_ in small‐breed dogs

Median (range) TPA_POST_ did not differ between CCWO_CORA3_ (5.00°; range: 5.00° to 5.00°) and CCWO_CORA5_ (5.00°; range: 5.00° to 5.00°) surgeries (*p* = 1.000; rank biserial 0.05, a small effect), but median MAA was less in CCWO_CORA3_ (3.00°; 3.00° to 3.00°) than in CCWO_CORA5_ (5.00°; range: 5.00° to 5.00°). There was no difference in median *Δ*TL between CCWO_CORA3_ planned surgeries (+1.37%; +0.75% to +1.90%) and for CCWO_CORA5_ planned surgeries (+1.29%; +1.01% to +1.67%; *p* = 1.00; rank biserial 0.05, a small effect). However, median wedge angle differed between CCWO_CORA3_ (30.5°; range: 29.7° to 32.7°) and CCWO_CORA5_ (32.5°; range: 31.7° to 34.7°) surgeries (*p* = .031; rank biserial 1.00, a large effect).

## 4 DISCUSSION

This study demonstrated that, compared with other methods, the CCWO_CORA_ methodology achieved an exact target TPA_POST_, with much less variability, across all tibial morphologies. This suggests that this new approach is more predictable than existing methods.

The MAA must occur during CCWO irrespective of the planning methodology.^25^ Most CCWO surgeries follow Paley's third rule of osteotomy correction, namely that angular correction is achieved through translation and failure to align the proximal and distal mechanical/anatomical axes.^26^ This translation will have an unpredictable and varying magnitude of effect on the MAA, as evidenced by the wider coefficient of variation (COV) seen in this study. Increasing MAA diminishes the effect of a defined wedge angle on TPA_POST_ and, therefore, any technique that induces more MAA requires a larger wedge angle to achieve target TPA_POST_. In a recent meta‐analysis, the reported prediction interval for MAA ranged from 2.5° to 5.4° (mean of 3.9°).^27^ Based upon these findings, the MAA was set at 3° for all surgeries in the current study.

A TPA_POST_ ranging from 3.8° to 5.9° eliminates cranial tibial subluxation ex vivo.^10^ Given that an angle of 5° is generally accepted as the target TPA_POST_ for tibial plateau‐leveling procedures, this was selected as the target TPA_POST_ in the current study.^28^ The predictability of the CCWO_CORA_ technique is a direct consequence of defining the desired MAA and TPA_POST_ allowing application of CORA methodology. Plotting a PMA (based on target TPA_POST_) and DMA (based on desired MAA) defines the precise location and magnitude of the CORA. In doing so, the location of the wedge apex and the exact wedge angle required to achieve the target TPA_POST_ could be accurately calculated.

A 3° MAA and a 5° target TPA_POST_ were maintained to allow comparison across techniques and tibial morphologies. The main advantage of the described CCWO_CORA_ technique is that these parameters are selectable depending on surgeon preference and tibial morphology. Some authors have argued that a TPA_POST_ of 10° is more suitable for a tibial plateau‐leveling procedure,^16^ achieved by defining PMA at 80° rather than the 85° used in this study. Similarly, if a 3° TPA_POST_ were desired, then the PMA could be defined at 87°.

Certain tibial morphologies may require a greater MAA to execute the surgery successfully. In the current study, although a 3° MAA was technically possible in small‐breed tibial morphologies, the resultant ostectomy was deemed too proximal and the resultant bone stock too limited to allow safe bone‐plate application. This finding is unsurprising given the link between MAA and TPA; indeed, the mean MAA for large‐breed dogs was 2.6°, compared with 4.31° for small‐breed dogs with the other CCWO methodologies. By increasing the MAA from 3° to 5° in the small‐breed group, a more feasible CCWO_CORA_ was possible without affecting surgical accuracy.

The effects of mechanical axis advancement on stifle mechanics following tibial plateau altering osteotomy procedures have not been investigated but the effects of tibial anatomical‐mechanical axis angles (AMA‐angles) have.^29^, ^30^ Postoperatively, differences between the anatomical axis and mechanical axis resulting in an AMA‐angle of greater than 3° results in increased caudal displacement of the weightbearing axis, which causes a focal increase in joint forces at the caudal aspect of the tibial plateau.^30^ The CCWO_CORA_ methodology allows the surgeon to align the anatomical axis and mechanical axis, which allays concerns regarding a caudally orientated force, although the effects of transferring joint forces cranially have not been investigated.

Similarly, wedge orientation can be modified to facilitate technical execution and proximal fragment bone stock. In the current study, the proximal osteotomy of the wedge exited 5 mm distal to the patellar tendon insertion to maintain consistency across techniques. This resulted in osteotomies of unequal lengths for the CCWO_CORA_ surgeries. The cranial cortices were, therefore, not aligned as doing so would have caused Paley's third rule of osteotomy correction to apply, preventing achievement of the target TPA_POST_. Alignment of the cranial cortices in the sagittal plane in vivo may be technically easier to assess in the operating theater. During preoperative planning, provided that the apex of the defined wedge remains at the calculated ACA‐CORA, the wedge can be rotated until osteotomies of equal length are achieved, which will allow the cranial cortices to be aligned, while still obeying Paley's first rule of osteotomy correction; angular correction and alignment of the proximal and distal axes is achieved. If this adjustment is not made, surgeons should ensure that sagittal alignment is determined at the level of the apex of the wedge.

Previous reports have raised concerns over tibial shortening following CCWO.^32^, ^33^, ^34^ In the current study, change in tibial length was ±1%, depending on technique used, with CCWO_CORA_ and CCWO_ISO_ increasing tibial length and CORA_TPA_ and CORA_TPA‐5_ decreasing tibial length. A theoretical advantage of CCWO_CORA_ and CCWO_ISO_ is that tibial length is maintained but, clinically, the tibial shortening reported is likely to be clinically insignificant, being less than the 6% to 20% decrease in limb length that is tolerated following acute limb shortening.^35^, ^36^, ^37^

In silico surgical planning is commonly used across human surgical disciplines and, more recently, has also been used as the basis for CCWO planning in dogs.^7^ This approach offers multiple advantages over cadaveric studies. More surgeries can be performed and multiple surgeries can be performed on the same bone, which is advantageous when making case–control comparisons. It also avoids both the expense and ethical concerns resulting from the use of cadaveric specimens, while enabling exact surgical planning and execution that would not be replicable even in the hands of an experienced surgeon. This is particularly useful in proof‐of‐concept studies where a geometric principle is being investigated.

As with the TPLO procedure, CCWO_CORA_ methodology can determine the exact correction required to achieve the target TPA_POST_. Cranial closing wedge ostectomy does offer some advantages over the TPLO procedure. First, there is no reliance on specialized equipment,^9^ making the surgery potentially more accessible. The TPLO procedure has been demonstrated as a safe technique to stabilize cranial cruciate ligament‐deficient stifles with excessive TPA,^38^ CCWO may be more suited to dogs with this conformation, avoiding the need for substantial rotation of the proximal bone fragment. The CCWO_CORA_ methodology described is well suited for the treatment of concurrent torsional, varus, or valgus tibial deformities.^11^ This could be facilitated by in silico planning and the use of 3D‐printed surgical guides to improve accuracy.^39^

The main limitation of this study is the ex vivo, in silico design. Although this approach ensures that conditions are well controlled, enabling perfect surgical planning and execution, the same degree of accuracy is unlikely to be achieved clinically. Following on from this proof‐of‐concept study, a prospective, randomized cohort study measuring the accuracy of TPA_POST_ of CCWO_CORA_, against another of the described CCWO methodologies, is now indicated. Whilst MAA is an inevitability of any CCWO procedure, its consequence on stifle biomechanics is not fully understood. Further research may focus on the impact of MAA on stifle stability and changes in stifle joint contact mechanics.

### 4.1 Conclusion

This novel CCWO_CORA_ is the first method to be described that can accurately and predictably achieve target TPA_POST_. Further studies are required to establish whether these results can be transferred to the clinical setting.

## AUTHOR CONTRIBUTIONS

Petchell WHR, BVMedSci, BVM, BVS, AFHEA, MRCVS: Contributed to the design of the study, identified suitable medical records, planned the in silico surgeries, interpreted data, and drafted and revised the manuscript. Bostock AR, BEng, MSc: Planned and performed all in silico surgeries to acquire data; provided scientific in‐line editing of the manuscript. German AJ, BVSc, PhD, CertSAM, DipECVIM‐CA, SFHEA, FRCVS: Performed statistical analysis of the data and provided scientific in‐line editing of the manuscript. Tomlinson AW, BVSc, CertAVP(GSAS), DipECVS, FHEA, MRCVS: Conceptualized the methodology, contributed the design of the study, identified suitable medical records, planned the in silico surgeries, and provided scientific in‐line editing of the manuscript. All authors provided a critical review of the manuscript and endorsed the final version. All authors are aware of their respective contributions and have confidence in the integrity of all contributions.

## FUNDING INFORMATION

The authors received no grants or financial support related to this report.

## CONFLICT OF INTEREST

Alexander J. German is an employee of the University of Liverpool, but his position is financially supported by Royal Canin. Alexander J. German has also received financial remuneration and gifts for providing educational material, speaking at conferences, and consultancy work, all unrelated to the current study. Anna R. Bostock is an employee of Fusion Implants who design and manufacture veterinary orthopedic implants. The authors declare no other potential conflicts of interest with respect to the research, authorship, and/or publication of this article. The authors declare no other commercial or financial relationships that could be construed as a potential conflict of interest with respect to the research, authorship and publication of this article.

## Data Availability

The data that support the findings of this study are available from the corresponding author upon reasonable request.

## References

[1] Slocum B , Devine T . Cranial tibial wedge osteotomy: a technique for eliminating cranial tibial thrust in cranial cruciate ligament repair. J Am Vet Med Assoc. 1984;184:564‐569. 6706801

[2] Corr SA , Brown C . A comparison of outcomes following tibial plateau levelling osteotomy and cranial tibial wedge osteotomy procedures. Vet Comp Orthop Traumatol. 2007;20:312‐319. 18038011 10.1160/vcot-07-02-0013

[3] Wallace AM , Addison ES , Smith BA , Radke H , Hobbs SJ . Modification of the cranial closing wedge ostectomy technique for the treatment of canine cruciate disease. Description and comparison with standard technique. Vet Comp Orthop Traumatol. 2011;24:457‐462. 21976135 10.3415/VCOT-10-11-0159

[4] Oxley B , Gemmill TJ , Renwick AR , Clements DN , McKee WM . Comparison of complication rates and clinical outcome between tibial plateau leveling osteotomy and a modified cranial closing wedge osteotomy for treatment of cranial cruciate ligament disease in dogs. Vet Surg. 2013;42:739‐750. 23889810 10.1111/j.1532-950X.2013.12033.x

[5] Frederick SW , Cross AR . Modified cranial closing wedge osteotomy for treatment of cranial cruciate ligament insufficiency in dogs with excessive tibial plateau angles: technique and complications in 19 cases. Vet Surg. 2017;46:403‐411. 28145568 10.1111/vsu.12614

[6] Terreros A , Daye RM . Modified cranial closing wedge osteotomy to treat cranial cruciate ligament deficient stifles with excessive tibial plateau angles: complications, owner satisfaction, and midterm to long‐term outcomes. Vet Surg. 2020;49:1109‐1117. 32529724 10.1111/vsu.13431

[7] Banks C , Jones GMC , Meeson RL . A mismatch of planning and achieved tibial plateau angle in cranial closing wedge surgery: an in silico and clinical evaluation of 100 cases. Vet Surg. 2024;53:113‐121. 37470173 10.1111/vsu.13998

[8] Slocum B , Slocum TD . Tibial plateau leveling osteotomy for repair of cranial cruciate ligament rupture in the canine. Vet Clin N Am Small Anim Pract. 1993;23:777‐795. 10.1016/s0195-5616(93)50082-7 8337790

[9] Holsworth IG . Clinical comparison of TPLO vs tibial closing wedge osteotomy. Proceedings, 12th European Society of Veterinary Orthopaedics and Traumatology; 2004.

[10] Apelt D , Pozzi A , Marcellin‐Little DJ , Kowaleski MP . Effect of cranial tibial closing wedge angle on tibial subluxation: an ex vivo study. Vet Surg. 2010;39:454‐459. 20345522 10.1111/j.1532-950X.2010.00670.x

[11] Kim SE , Pozzi A , Kowaleski MP , et al. Tibial osteotomies for cranial cruciate ligament insufficiency in dogs. Vet Surg. 2008;37:111‐125. 18251804 10.1111/j.1532-950X.2007.00361.x

[12] Paley D , Herzenberg JE , Tetsworth K , McKie J , Bhave A . Deformity planning for frontal and sagittal plane corrective osteotomies. Orthop Clin North Am. 1994;25:425‐465. 8028886

[13] Knapp JL , Tomlinson JL , Fox DB . Classification of angular limb deformities affecting the canine radius and ulna using the Center of Rotation of angulation method. Vet Surg. 2016;45:295‐302. 27011252 10.1111/vsu.12460

[14] Fox DB , Tomlinson JL , Cook JL , et al. Principles of uniapical and biapical radial deformity correction using dome osteotomies and the center of rotation of angulation methodology in dogs. Vet Surg. 2006;35:67‐77. 16409412 10.1111/j.1532-950X.2005.00114.x

[15] Dismukes DI , Fox DB , Tomlinson JL , Essman SC . Use of radiographic measures and three‐dimensional computed tomographic imaging in surgical correction of an antebrachial deformity in a dog. J Am Vet Med Assoc. 2008;232:68‐73. 18167111 10.2460/javma.232.1.68

[16] Raske M , Hulse D , Beale B , Saunders WB , Kishi E , Kunze C . Stabilization of the CORA based leveling osteotomy for treatment of cranial cruciate ligament injury using a bone plate augmented with a headless compression screw. Vet Surg. 2013;42:759‐764. 23876155 10.1111/j.1532-950X.2013.12035.x

[17] Slocum B , Devine T . Cranial tibial thrust: a primary force in the canine stifle. J Am Vet Med Assoc. 1983;183:456‐459. 6618973

[18] Morris E , Lipowitz AJ . Comparison of tibial plateau angles in dogs with and without cranial cruciate ligament injuries. J Am Vet Med Assoc. 2001;218:363‐366. 11201561 10.2460/javma.2001.218.363

[19] Team RC . R: A Language and Environment for Statistical Computing. R Foundation for Statistical Computing; 2023.

[20] Lesnoff M , Lancelot R . aod: Analysis of Overdispersed Data. 2012 http://cran.r-project.org/package=aod.

[21] Pinheiro J . BD: _nlme: linear and nonlinear mixed effects Models. Team R, https://CRAN.R-project.org/package=nlme.; 2024.

[22] Lenth R . emmeans: Estimated Marginal Means, aka Least‐Squares Means, R package version 1.8.7. https://CRAN.R-project.org/package=emmeans 2024.

[23] Lakens D . Calculating and reporting effect sizes to facilitate cumulative science: a practical primer for t‐tests and ANOVAs. Front Psychol. 2013;4(863). 10.3389/fpsyg.2013.00863 PMC3840331 24324449

[24] Funder DC , Ozer DJ . Evaluating effect size in psychological research: sense and nonsense. Adv Methods Pract Psychol Sci. 2019;2(2):156‐168.

[25] Moreira LR , Sparks T , Ogden DM . Predicting tibial plateau angles following four different types of cranial closing wedge ostectomy. Vet Surg. 2024;53:143‐154. 37749853 10.1111/vsu.14033

[26] D P . Principles of Deformity Correction. Springer; 2002.

[27] Miles JE , Nielsen MBM . Reported accuracy of cranial closing wedge ostectomy variants for management of canine cranial cruciate ligament insufficiency: a systematic review and meta‐analysis. Vet J. 2023;295:105989. 37148995 10.1016/j.tvjl.2023.105989

[28] Dejardin L . Tibial plateau levelling osteotomy. In: Slatter D , ed. Textbook of Small Animal Surgery. Saunders; 2003.

[29] Guenego L , Payot M , Charru P , et al. Comparison of tibial anatomical‐mechanical axis angle between predisposed dogs and dogs at low risk for cranial cruciate ligament rupture. Vet J. 2017;225:35‐41. 28720297 10.1016/j.tvjl.2017.04.011

[30] Guénégo L , Vezzoni A , Vezzoni L . Comparison of tibial anatomical‐mechanical axis angles and patellar positions between tibial plateau levelling osteotomy (TPLO) and modified cranial closing wedge osteotomy (AMA‐based CCWO) for the treatment of cranial cruciate ligament disease in large dogs with tibial plateau slopes greater than 30° and clinically normal Labradors retrievers. BMC Vet Res. 2021;17:368. 34861875 10.1186/s12917-021-03094-3 PMC8641203

[31] Kim SE , Pozzi A , Banks SA , et al. Effect of tibial plateau leveling osteotomy on femorotibial contact mechanics and stifle kinematics. Vet Surg. 2009;38:23‐32. 19152614 10.1111/j.1532-950X.2008.00470.x

[32] Campbell KA , Payne JT , Doornink MT , Haggerty J . Outcome of tibial closing wedge osteotomy in 55 cranial cruciate ligament‐deficient stifles of small dogs (< 15 kg). Vet Surg. 2016;45:1056‐1062. 27804139 10.1111/vsu.12558

[33] Christ JP , Anderson JR , Youk AO . Modified cranial closing wedge ostectomy in 25 dogs. Vet Surg. 2018;47:683‐691. 30129063 10.1111/vsu.12912

[34] Lee J , Kim D , Oh H , Lee S , Choi SH , Kim G . Radiographic comparison of cranial tibial wedge osteotomy versus tibial plateau leveling osteotomy: a cadaveric study. J Veterinary Clinics. 2022;39:93‐99.

[35] Franczuszki D . The post‐operative effects of femur shortening in the mature dog. 1986.

[36] Boston SE , Skinner OT . Limb shortening as a strategy for limb sparing treatment of appendicular osteosarcoma of the distal radius in a dog. Vet Surg. 2018;47:136‐145. 28990681 10.1111/vsu.12726

[37] Andrews C , Williams R , Burneko M . Use of liposomal bupivacaine in dogs and cats undergoing gastrointestinal surgery is not associated with a higher rate of surgical site infections or multidrug‐resistant infections. J Am Vet Med Assoc. 2024;262:1‐6. 10.2460/javma.23.08.0463 37918106

[38] Barnes DC , Trinterud T , Owen MR , Bush MA . Short‐term outcome and complications of TPLO using anatomically contoured locking compression plates in small/medium‐breed dogs with “excessive” tibial plateau angle. J Small Anim Pract. 2016;57:305‐310. 27148864 10.1111/jsap.12486

[39] de Armond CC , Lewis DD , Townsend S . Use of preoperative 3D virtual planning and 3D‐printed patient‐specific guides to facilitate a single‐stage cranial closing wedge ostectomy and tibial plateau leveling osteotomy procedure to address proximal tibial deformity, an excessive tibial plateau angle, and cranial cruciate ligament insufficiency in a dog. Case Rep Vet Med. 2023;2023:3368794. 38045562 10.1155/2023/3368794 PMC10689072

